# TRMT10A regulates tRNA-ArgCCT m^1^G9 modification to generate tRNA-derived fragments influencing vasculogenic mimicry formation in glioblastoma

**DOI:** 10.1038/s41419-025-07548-6

**Published:** 2025-03-26

**Authors:** Deng Wei, Bei Zhai, Hui Zeng, Long Liu, Han Gao, Shiqi Xiang, Xiaobai Liu, Jun Ma, Yang Lin, Yilong Yao, Ping Wang

**Affiliations:** 1 Department of Neurobiology, School of Life Sciences, China Medical University, Shenyang, China; 2 Department of Neurosurgery, Shengjing Hospital of China Medical University, Shenyang, China

**Keywords:** Small RNAs, CNS cancer

## Abstract

Glioblastoma multiforme (GBM) is the most common and aggressive primary central nervous system tumor. The formation of vasculogenic mimicry (VM) in GBM is closely related to poor patient prognosis. Therefore, it is urgently necessary to explore the mechanisms that promote VM formation in GBM and identify therapeutic targets. CGGA data analysis revealed that TRMT10A expression is significantly downregulated in WHO grade IV primary glioma samples compared to grade II samples, consistent with the protein expression levels. Additionally, GBM patients with low TRMT10A expression have poorer prognoses. In human glioma cells, TRMT10A expression is significantly lower than in human astrocytes. Knockdown of TRMT10A reduces m^1^G9 modification of tRNA-ArgCCT, upregulates tRF-22 expression, and promotes glioma cell proliferation, migration, invasion, and tube formation. Overexpression of tRF-22 in glioma cells significantly downregulates MXD1 expression. tRF-22 negatively regulates MXD1 expression by binding to its 3’UTR, reducing MXD1’s transcriptional inhibition of HIF1A, thereby promoting glioma cell proliferation, migration, invasion, and tube formation. Overexpression of TRMT10A combined with tRF-22 inhibition significantly reduces the number of VM channels and inhibits tumor growth in xenograft models in nude mice. This study elucidates the mechanism by which TRMT10A affects VM formation in glioma and provides a novel therapeutic target for GBM.

## Introduction

Glioblastoma (GBM) is the most malignant primary central nervous system tumor, with a poor prognosis [1]. Despite surgery, radiotherapy, and chemotherapy, the five-year relative survival rate for GBM patients remains at 6.8%, the lowest among malignant brain tumors. Vasculogenic mimicry (VM) is a blood supply system formed by tumor cells and basement membrane that connects to host microcirculation, independent of endothelial cells, and is closely linked to GBM’s poor prognosis [2]. Understanding the mechanisms of VM formation and identifying targeted therapies are crucial for improving GBM patient outcomes.

Recent evidence suggests non-coding RNAs play crucial roles in tumor growth regulation [3]. Transfer RNA (tRNA), a highly expressed non-coding RNA, recognizes mRNA codons via its anticodon and transports amino acids for peptide chain synthesis on ribosomes. Studies confirm tRNA modifications are associated with tumors, neurological, and metabolic disorders [4, 5]. Post-transcriptional tRNA modifications, particularly methylation, enhance tRNA structural stability, proper folding, and reduce translation errors [6]. The TRMT10 family, including TRMT10A, TRMT10B, and TRMT10C, catalyzes the 1-methylguanosine (m^1^G) modification at tRNA position 9. TRMT10A, significantly more efficient than TRMT10B, primarily regulates m^1^G9 modification of tRNA-Arg and tRNA-Trp [7]. Loss-of-function mutations in TRMT10A cause early-onset diabetes, microcephaly, and other diseases [8, 9]. CGGA data show TRMT10A is significantly downregulated in WHO grade IV gliomas compared to grade II, correlating with poor prognosis. The role of TRMT10A in glioma VM formation remains unexplored.

The reduction in tRNA methyltransferase activity leads to hypomethylated tRNAs, making them susceptible to nuclease-induced degradation or fragmentation, and promoting the generation of tRNA halves (tiRNAs) or tRNA-derived fragments (tRFs) [10, 11]. These tRFs are categorized as tRF-1, tRF-2, tRF-3, tRF-5, and inter-tRF (i-tRF) based on their cleavage sites. Hypomethylated tRNAs with abnormally expressed tRFs have been found in various cancers, affecting protein translation [12], gene expression [13, 14], and cell cycle regulation [15]. Differentially expressed tRFs can serve as biomarkers for cancer diagnosis or prognosis and potential therapeutic targets [16]. tRFs can function like miRNAs by binding to Ago proteins or regulating mRNA stability through RNA-binding proteins [17, 18]. For example, tRF-T11, derived from the 5’ end of tRNA-HisGUG, interacts with AGO2 in ovarian cancer cells, targeting the 3’UTR of TRPA1 and inhibiting its expression [19].

Our preliminary work demonstrated that downregulating TRMT10A in U251 glioma cells led to the upregulation of differentially expressed tRFs, with tRF-22-8XF6RE98N (tRF-22) showing the most significant increase. tRF-22 is derived from tRNA-ArgCCT. Further analysis predicted a potential binding site for tRF-22 in the 3’UTR of MAX dimerization protein 1 (MXD1) mRNA. MXD1, part of the MYC/MXD/MAX family, is a basic helix-loop-helix leucine zipper (bHLH-Zip) protein acting as a transcriptional repressor. It competes with MYC for MAX binding by recognizing the E-box sequence 5’-CAC[GA]TG-3’, forming a transcriptional repressor complex that inhibits cell differentiation, proliferation, and apoptosis [20, 21]. MXD1 downregulation is associated with the enhancement of proliferation, invasion, and metastatic potential in pancreatic, breast, and gastric cancer cells [22–24]. HIF-1α promotes VM formation in various tumors, including lung adenocarcinoma, cervical cancer, and liver cancer, by regulating the expression of VM-related molecules [25–27]. Analysis of the HIF1A gene promoter region revealed the presence of the MXD1 recognition motif 5’-CACGTG-3’, suggesting MXD1 may negatively regulate HIF-1α expression and modulate VM formation in gliomas.

This study confirmed that downregulation of TRMT10A in glioma cells reduces m^1^G9 modification of tRNA-ArgCCT, decreasing tRNA stability and upregulating tRF-22. tRF-22 negatively regulates the transcription factor MXD1 expression, diminishing its transcriptional repression of the HIF1A gene, thus promoting VM formation in gliomas. Overexpression of TRMT10A and inhibition of tRF-22 significantly reduces xenograft tumor size and VM formation in nude mice. This study elucidates the mechanism by which TRMT10A affects VM formation in gliomas and provides a novel therapeutic target for GBM.

## Materials and methods

### Patient tissue samples, cell culture and cell transfection

Details on the tissue specimens, cell lines used in this study, and the protocols for cell transfection experiments are presented in the Supplementary Methods. All the sequences are shown in Supplementary Tables 1 and 2.

### tRFs and tiRNAs sequencing and data analysis

After knocking down TRMT10A in U251 cells, the knockdown group and the NC group cells were collected for tRFs and tiRNAs sequencing. The sequencing libraries were absolutely quantified using an Agilent BioAnalyzer 2100. Sequencing analysis was performed via the Illumina NextSeq 500 platform (Aksomics, Shanghai, China) according to the manufacturer’s protocol. The tRFs and tiRNAs sequencing analysis were performed using the Arraystar tRF and tiRNA-seq data package.

### Reverse transcription and quantitative real-time polymerase chain reaction (RT-qPCR)

Total RNA was extracted from cells using Trizol reagent (Life Technologies, CA, USA) according to the manufacturer’s instructions. RT-qPCR was performed using the PrimeScript™ II 1st Strand cDNA Synthesis Kit and TB Green^®^ Premix Ex Taq™ II (Tli RNaseH Plus, Takara, Japan). The tRNA-ArgCCT was detected as previously described [28]. The primers used above are listed in Supplementary Table 3. For further details, please refer to the Supplementary Methods.

### Western blot and dot blot analysis

Detailed descriptions of the Western blot and dot blot analysis can be found in the Supplementary Methods. The primary antibodies used are listed in Supplementary Table 4.

### Chromatin immunoprecipitation (ChIP)-qPCR assay

ChIP analysis was performed using a chromatin immunoprecipitation assay kit (Cell Signaling Technology, MA, USA). The procedure for ChIP has been described previously [29]. For further details, please refer to the Supplementary Methods. The antibody and primer sequences used are shown in Supplementary Tables 4–6.

### Nuclear and cytoplasmic RNA extraction

The RNA extraction was performed according to the instructions provided with the nuclear-cytoplasmic separation kit (Norgen Biotek, Canada). For further details, please refer to the Supplementary Methods.

### RNA fluorescence in situ hybridization (FISH) and immunofluorescence (IF)

The details of the FISH and IF analyses are provided in the Supplementary Methods. The primary antibodies used are listed in Supplementary Table 4.

### Subcutaneous and orthotopic xenograft tumor model in nude mice

Six-week-old BALB/c female nude mice were obtained from Huafukang Company (Beijing, China) and divided into the following four groups: OE-NC + sh-NC, OE-TRMT10A, sh-tRF-22, OE-TRMT10A + sh-tRF-22, with 5 mice in each group. For subcutaneous xenograft tumor model, the procedure has been described previously [29]. For the orthotopic xenograft tumor model, glioma cells stably overexpressing TRMT10A or with reduced expression of tRF-22 were labeled with the Luc reporter gene using a lentiviral vector. 5 × 10^4^ cells in 3 µL of PBS were stereotactically injected into the right striatum of 6-week-old BALB/c nude mice (coordinates relative to the bregma: medial-lateral +2 mm, anterior-posterior +1 mm and dorsal-ventral −3 mm). Tumor growth was monitored using an in vivo imaging system (SkyView, China). A region of interest was defined over the tumors to quantify signal intensities recorded as total photon counts per second per cm^2^ (photons/sec/cm^2^/sr). All nude mouse experiments were conducted strictly in accordance with the approved protocol by the Ethics Committee of China Medical University (KT2022330).

### CD31-periodic acid-Schiff (PAS) and immunohistochemistry (IHC) staining

The procedure for CD31-PAS and IHC staining has been described previously [29]. For further details, please refer to the Supplementary Methods. The primary antibodies used are listed in Supplementary Table 4.

### Statistical analysis

Results are presented as mean ± standard deviation. GraphPad Prism 8.0 was used for data analysis. The unpaired *t*-test was used for intergroup difference analysis, and one-way ANOVA was used for analysis of differences among multiple groups caused by single-factor variation. A *P*-value < 0.05 was considered statistically significant.

## Results

### Downregulated TRMT10A promotes VM formation in glioma cells

**Fig. 1:**
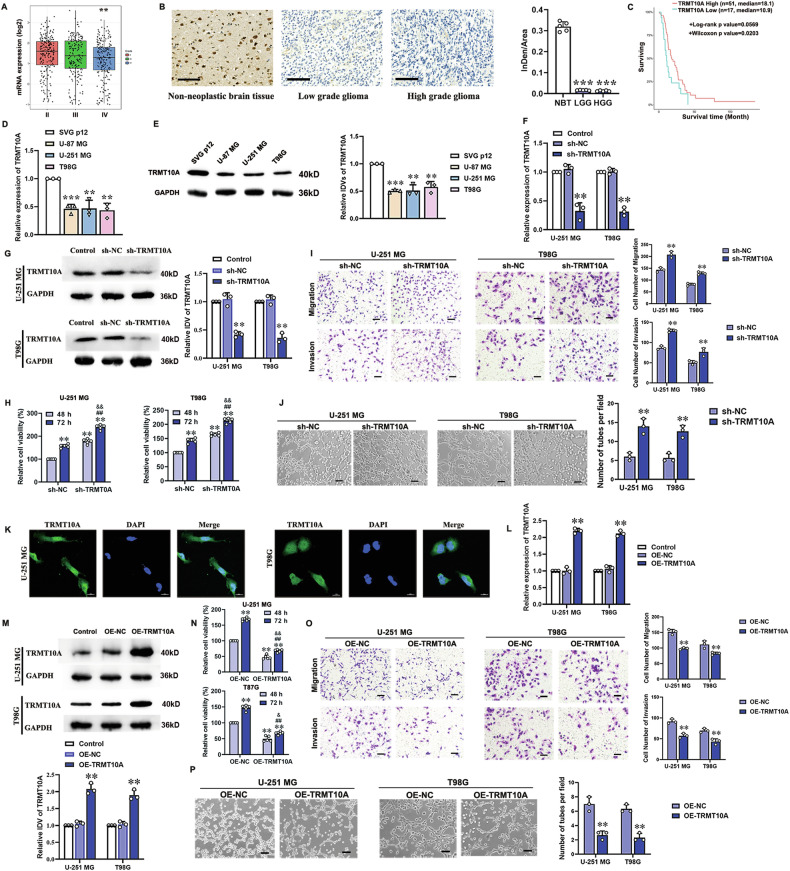
Downregulation of TRMT10A in glioma cells promotes VM formation. **A** The mRNA expression levels of TRMT10A in WHO grade II–IV glioma samples were analyzed using the CGGA database. Values are presented as mean ± SD, **P < 0.01 compared to WHO grade II. **B** Representative images of TRMT10A immunohistochemical stains in peritumoral non-glioma tissue, low-grade glioma (LGG) and high-grade glioma (HGG). Values are presented as mean ± SD (*n* = 5), ***P < 0.001 compared to non-neoplastic brain tissue. Scale bar = 100 μm. **C** The effect of TRMT10A expression levels on the survival of GBM patients was analyzed using the CGGA database. The expressions of TRMT10A in human astrocyte cell line SVG p12, and glioma cell lines U-87 MG, U-251 MG, and T98G were detected by RT-qPCR (**D**) and Western blot (**E**), respectively. Values are presented as mean ± SD (*n* = 3), **P < 0.01, ***P < 0.001 compared to the SVG p12 cell group. The TRMT10A knockdown levels in U-251 MG and T98G glioma cells were verified by RT-qPCR (**F**) and western blot (**G**), respectively, following the transfection of sh-TRMT10A vectors. After 48 h and 72 h of culture with stable TRMT10A knockdown U-251 MG and T98G cells, **H** cell proliferation was assessed using the CCK-8 assay (*n* = 5). Values are presented as mean ± SD, **P < 0.01 compared to the sh-NC group of 48 h; ^##^*P* < 0.01 compared to the sh-NC group of 72 h; ^&&^*P* < 0.01 compared to the sh-TRMT10A group of 48 h. **I** Cell migration and invasion abilities were evaluated using the Transwell assay (*n* = 3). **J** The tube formation ability of cells was assessed using the tube formation assay (*n* = 3). Values are presented as mean ± SD, **P < 0.01 compared to the sh-NC group. Scale bar = 50 μm. **K** Subcellular localization of TRMT10A in U-251 MG and T98G cells was observed using laser confocal microscopy. Scale bar = 20 μm. The expressions of TRMT10A in U-251 MG and T98G cells after transfection with TRMT10A overexpression vectors were verified by RT-qPCR (**L**) and western blot (**M**), respectively. Values are presented as mean ± SD (*n* = 3), **P < 0.01 compared to the OE-NC group. After 48 h and 72 h of culture with stable TRMT10A overexpression in U-251 MG and T98G cells, **N** cell proliferation was assessed using the CCK-8 assay (*n* = 5). Values are presented as mean ± SD, **P < 0.01 compared to the sh-NC group of 48 h; ^##^*P* < 0.01 compared to the sh-NC group of 72 h; ^&^*P* < 0.05, ^&&^*P* *<* 0.01 compared to the sh-TRMT10A group of 48 h. **O** Cell migration and invasion abilities were evaluated using the Transwell assay (*n* = 3). Scale bar = 50 μm. **P** The tube formation ability of cells was assessed using the tube formation assay (*n* = 3). Values are presented as mean ± SD, **P < 0.01 compared to the OE-NC group. Scale bar = 50 μm.

Analysis of CGGA data revealed that TRMT10A expression was significantly lower in WHO grade IV primary glioma samples compared to WHO grade II samples (Fig. 1A). Immunohistochemistry staining revealed that TRMT10A, mainly as cytoplasmic staining was found to be highly expressed in non-neoplastic brain tissues, while no strong immunoreactivity was detected in glioma tissues of low-grade (LGG, WHO I-II) and high-grade (HGG, WHO III-IV) (Fig. 1B). GBM patients with low TRMT10A expression had poorer prognoses (Fig. 1C). The mRNA and protein levels of TRMT10A were lower in glioma cells (U-87 MG, U-251 MG, T98G) compared to human astrocytes (SVG p12) (Fig. 1D, E). Further, TRMT10A was knocked down in U-251 MG and T98G cells using sh-TRMT10A, confirmed by RT-qPCR and Western blot, showing significant reduction in TRMT10A expressions (Fig. 1F, G). TRMT10A downregulation significantly increased U-251 MG and T98G glioma cell viability (Fig. 1H), migration, invasion (Fig. 1I), and tube formation (Fig. 1J) compared to sh-NC cells.

In U-251 MG and T98G cells, TRMT10A is primarily distributed in the cytoplasm (Fig. 1K). TRMT10A expression was upregulated in U-251 MG and T98G cells by transfecting OE-TRMT10A, confirmed by RT-qPCR and Western blot, showing significant increases compared to the OE-NC group (Fig. 1L, M). Upregulated TRMT10A significantly decreased glioma cell viability (Fig. 1N), migration, invasion (Fig. 1O), and tube formation (Fig. 1P) compared to OE-NC cells. These findings indicate that downregulation of TRMT10A promotes VM formation in glioma cells.

### TRMT10A regulates m^1^G9 modification of tRNA-ArgCCT, affecting tRNA-ArgCCT and tRF-22 expression

**Fig. 2:**
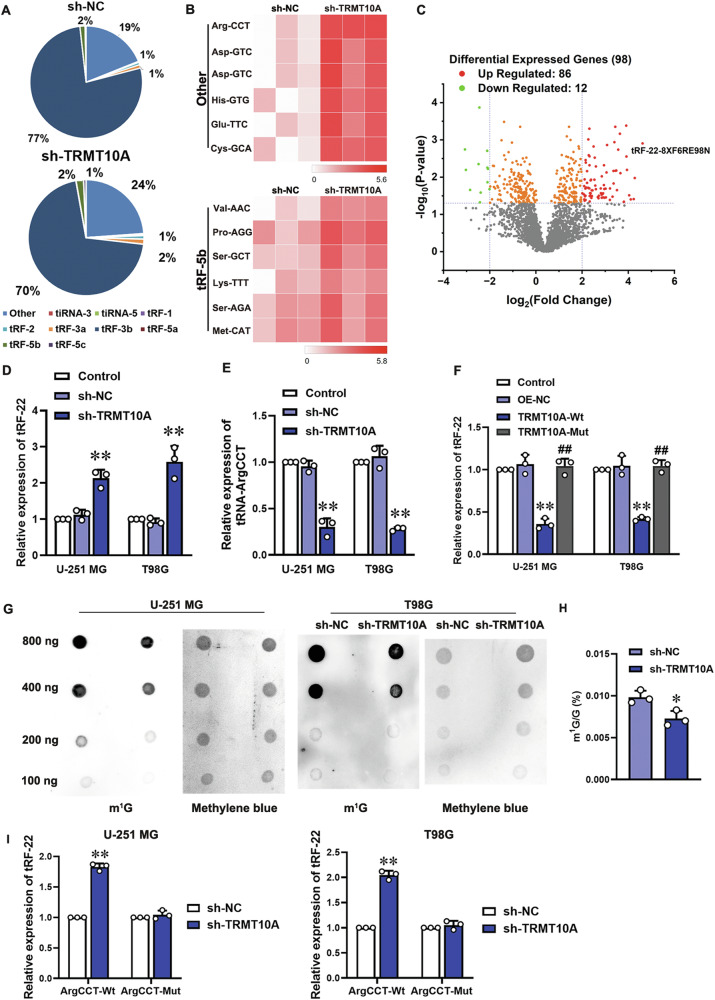
TRMT10A promotes m^1^G9 modification of tRNA-ArgCCT, regulating the expression of tRNA-ArgCCT and tRF-22. **A** Distributions of different subtypes of tRFs and tiRNAs in sh-NC and sh-TRMT10A groups from human tRF & tiRNA sequencing. **B** Sequencing analysis of the top two highly differentially expressed tRFs and tiRNAs following TRMT10A knockdown in U-251 MG cells. **C** Volcano plot showed differentially expressed tRFs and tiRNAs following TRMT10A knockdown in U-251 MG cells. (|log2FC| > 2, *P* < 0.05) 86 tRFs and tiRNAs upregulated (red) and 12 downregulated (green). The expression levels of tRF-22 (**D**) and tRNA-ArgCCT (**E**) in TRMT10A knockdown U-251 MG and T98G cells were detected by RT-qPCR. Values are presented as mean ± SD (*n* = 3), **P < 0.01 compared to the sh-NC group. **F** The expression changes of tRF-22 in U-251 MG and T98G cells overexpressing either wild-type TRMT10A (TRMT10A-Wt) or catalytically inactive mutant TRMT10A (Mut/G206R) were detected by RT-qPCR. Values are presented as mean ± SD (*n* = 3), **P < 0.01 compared to the OE-NC group; ^##^*P* < 0.01 compared to the TRMT10A-Wt group. **G** m^1^G dot blot analysis was used to assess changes in the overall m^1^G modification levels of total RNA in TRMT10A knockdown U-251 MG and T98G cells. **H** RNA pull-down coupled with LC-MS analysis was used to determine the m^1^G modification levels of tRNA-ArgCCT in TRMT10A knockdown U-251 MG cells. Values are presented as mean ± SD, *P < 0.05 compared to the sh-NC group. **I** The expression changes of tRF-22 in TRMT10A knockdown U-251 MG and T98G cells overexpressing either wild-type tRNA-ArgCCT (tRNA-ArgCCT-Wt) or a G9U methylation site mutant (tRNA-ArgCCT-Mut) were detected by RT-qPCR. Values are presented as mean ± SD (*n* = 3), **P < 0.01 compared to the sh-NC group.

To investigate how TRMT10A promotes VM formation in glioma cells, we knocked down TRMT10A in U-251 MG cells and analyzed the differentially expressed tRFs using Human tRF & tiRNA sequencing. “Other” tRF subtypes were significantly upregulated, followed by tRF-5b (Fig. 2A, B). Knockdown of TRMT10A led to upregulation of 86 tRFs and tiRNAs and downregulation of 12 (Fig. 2C), with tRF-22-8XF6RE98N (tRF-22) showing the most significant increase. RT-qPCR confirmed tRF-22 upregulation in U-251 MG and T98G cells with TRMT10A knockdown (Fig. 2D). tRF-22, derived from tRNA-ArgCCT, was further investigated for its generation mechanism. tRNA-ArgCCT expression was downregulated in U-251 MG and T98G cells with TRMT10A knockdown (Fig. 2E). Overexpression of wild-type TRMT10A, but not the catalytically inactive mutant (G206R), reduced tRF-22 expression (Fig. 2F). m^1^G dot blot analysis showed reduced m^1^G methylation in total RNA of cells with TRMT10A knockdown (Fig. 2G). RNA pull-down combined with mass spectrometry using a biotin-labeled tRNA-Arg probe revealed reduced m^1^G methylation of tRNA-Arg in U-251 MG cells with TRMT10A knockdown (Fig. 2H). Moreover, overexpression of wild-type tRNA-ArgCCT, but not that of G9 methylation site mutant (G9U), increased tRF-22 expression in glioma cells with TRMT10A knockdown (Fig. 2I). These findings suggest that TRMT10A downregulation in glioma cells weakens m^1^G9 modification of tRNA-ArgCCT, reducing its expression and enhancing tRF-22 expression.

### TRMT10A influences VM formation by regulating tRF-22 expression

**Fig. 3:**
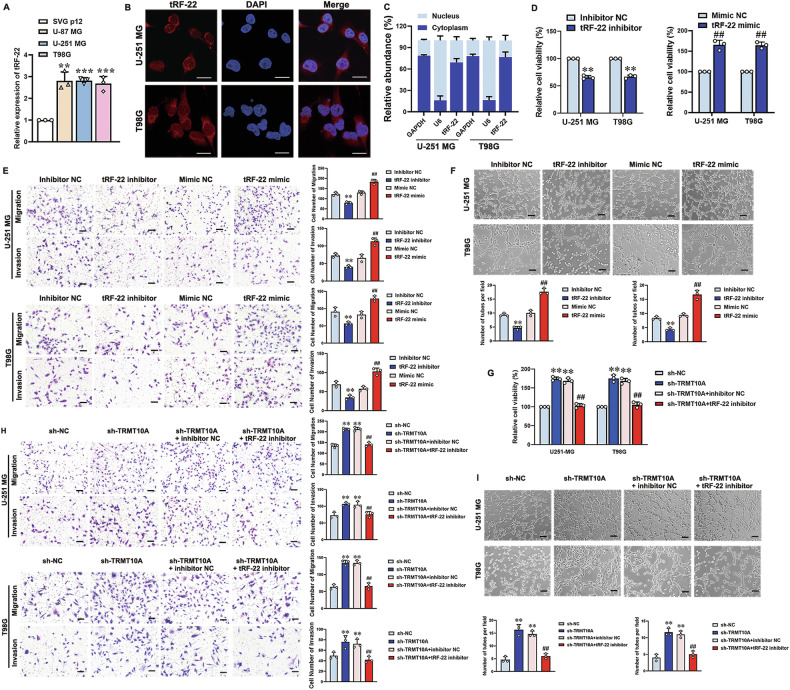
TRMT10A affects VM formation by regulating the expression of tRF-22. **A** The expressions of tRF-22 in SVG p12, U-87 MG, U-251 MG, and T98G cells were detected by RT-qPCR. Values are presented as mean ± SD (*n* = 3), **P < 0.01, ***P < 0.001 compared to the SVG p12 cell group. **B** Subcellular localization of tRF-22 in U-251 MG and T98G cells was observed using laser confocal microscopy. Scale bar = 20 μm. **C** After RNA nuclear-cytoplasmic separation, the expressions of tRF-22 in the nucleus and cytoplasm of U-251 MG and T98G cells was detected by RT-qPCR. GAPDH and U6 were used as cytoplasmic and nuclear RNA markers, respectively. **D** After 48 h of transfection with tRF-22 mimic and inhibitor in U-251 MG and T98G cells, cell proliferation was assessed using the CCK-8 assay (*n* = 5). **E** Cell migration and invasion abilities were evaluated using the Transwell assay (*n* = 3). Scale bar = 50 μm. **F** Tube formation ability of cells was assessed using the tube formation assay (*n* = 3). Values are presented as mean ± SD, **P < 0.01 compared to the inhibitor NC group, ^##^*P* < 0.01 compared to the mimic NC group. Scale bar = 50 μm. **G** After 48 h of transfection with tRF-22 inhibitor in TRMT10A stable knockdown U-251 MG and T98G cells, cell proliferation was assessed using the CCK-8 assay (*n* = 5). **H** Cell migration and invasion abilities were evaluated using the Transwell assay (n = 3). **I** Tube formation ability of cells was assessed using the tube formation assay (n = 3). Values are presented as mean ± SD, **P < 0.01 compared to the sh-NC group; ^##^*P* < 0.01 compared to the sh-TRMT10A + inhibitor NC group. Scale bar = 50 μm.

To investigate the role of tRF-22 in glioma cells, RT-qPCR analysis showed significantly higher tRF-22 expression in U-87 MG, U-251 MG, and T98G cells compared to SVG p12 cells (Fig. 3A). FISH analysis and RNA subcellular fractionation confirmed that tRF-22 is predominantly localized in the cytoplasm of U-251 MG and T98G cells (Fig. 3B, C). Transfection of tRF-22 inhibitor or mimic into U-251 MG and T98G cells was conducted to assess its impact on cell proliferation, migration, invasion, and tube formation. Compared to their respective NC groups, glioma cells in the tRF-22 inhibitor group showed significantly reduced cell viability (Fig. 3D), migration, invasion (Fig. 3E), and tube formation (Fig. 3F), whereas glioma cells in the tRF-22 mimic group showed significant increases in these functions (Fig. 3D–F). These results suggest that tRF-22 upregulation promotes VM formation in glioma cells.

To determine if TRMT10A influences VM formation by regulating tRF-22, a tRF-22 inhibitor was transfected into U-251 MG and T98G cells with stable TRMT10A knockdown. The tRF-22 inhibitor significantly reversed the increases in cell viability (Fig. 3G), migration, invasion (Fig. 3H), and tube formation abilities (Fig. 3I) induced by TRMT10A knockdown.

### tRF-22 binds to and negatively regulates MXD1 expression, modulating VM formation

**Fig. 4:**
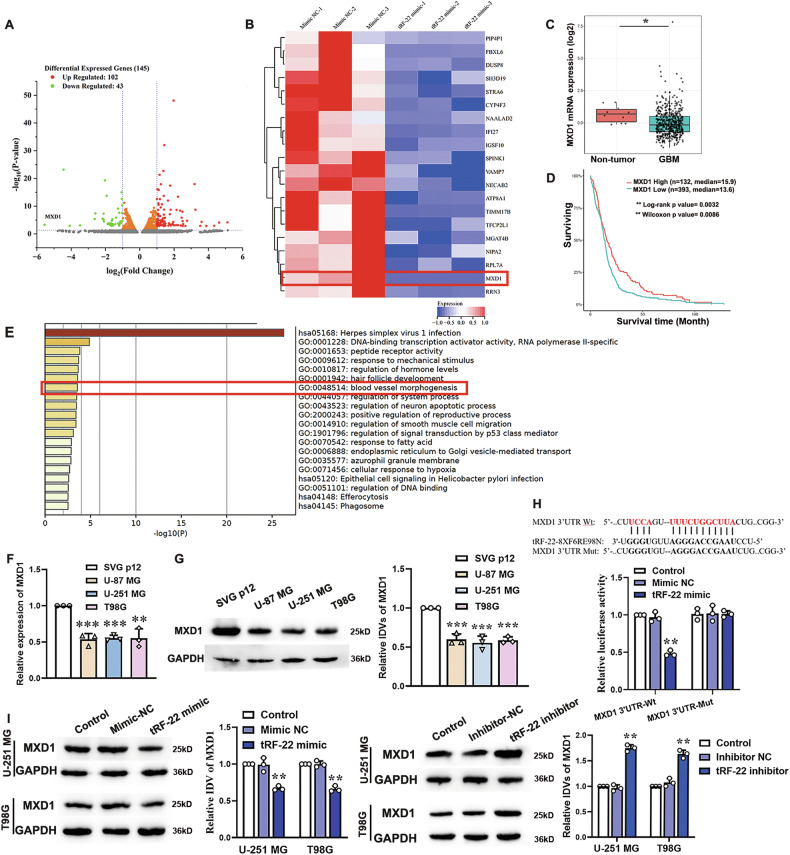
tRF-22 binds to and negatively regulates MXD1 expression. **A** Volcano plot showed differentially expressed genes following tRF-22 overexpression in U-251 MG cells. (|log2FC| > 2, *P* < 0.05) 102 genes upregulated (red) and 43 downregulated (green). **B** Hierarchical clustering heatmap of top 20 downregulated expressed mRNAs regulated by tRF-22 mimic. **C** The mRNA expression levels of MXD1 in non-tumor and GBM samples were analyzed using the TCGA database. Values are presented as mean ± SD, *P < 0.05 compared to non-tumor samples. **D** The effect of MXD1 expression levels on the survival of GBM patients was analyzed using the TCGA database. **E** The differentially expressed genes following tRF-22 overexpression were subjected to GO and KEGG enrichment, sorted by −log10(*P*). The expression levels of MXD1 in SVG p12, U-87 MG, U-251 MG, and T98G cells were detected by RT-qPCR (**F**) and western blot (**G**), respectively. Values are presented as mean ± SD (*n* = 3), **P < 0.01, ***P < 0.001 compared to the SVG p12 group. **H** The luciferase activity in 293T cells transfected with wild-type MXD1 3’UTR and tRF-22 binding site mutant dual-luciferase reporter vectors was analyzed using a dual-luciferase reporter system. Values are presented as mean ± SD (*n* = 5), **P < 0.01 compared to the mimic NC group. **I** After transfection with tRF-22 mimic and tRF-22 inhibitor in U-251 MG and T98G cells for 48 h, changes in MXD1 expression were detected by Western blot. Values are presented as mean ± SD (*n* = 3), **P < 0.01 compared to the NC group.

To explore the target genes regulated by tRF-22, tRF-22 mimic was transfected into U-251 MG cells. RNA-seq analysis revealed that transcriptome profiles in tRF-22-overexpressed cells were distinct from that of NC cells (Fig. 4A). The top 20 differentially expressed genes, ranked by LogFC values, were selected. Among the differentially expressed genes, MXD1 displayed the most significant down-regulation by tRF-22 overexpression (Fig. 4B). Analysis of TCGA data revealed that MXD1 expression was significantly lower in GBM samples compared to non-tumor samples (Fig. 4C). GBM patients with low MXD1 expression had poorer prognoses (Fig. 4D). Gene ontology (GO) analysis revealed that the differentially expressed genes were significantly enriched in blood vessel morphogeneisis (Fig. 4E). Furthermore, RT-qPCR and Western blot analyses were performed to assess MXD1 expressions in human astrocytes and glioma cells. Compared to SVG p12 cells, MXD1 mRNA and protein levels were significantly downregulated in U-87 MG, U-251 MG, and T98G cells (Fig. 4F, G).

To clarify the possible regulatory effects of tRF-22 on MXD1, dual-luciferase reporter assays showed that luciferase activity significantly decreased in the tRF-22 mimic and MXD1 3’UTR-Wt co-transfection group but remained unchanged in the tRF-22 mimic and MXD1 3’UTR-Mut co-transfection group (Fig. 4H). Western blot confirmed that MXD1 expression was significantly downregulated in glioma cells transfected with tRF-22 mimic and upregulated in those transfected with tRF-22 inhibitor (Fig. 4I). These findings indicate that tRF-22 binds to the MXD1 3’UTR and negatively regulates its expression in glioma cells.

**Fig. 5:**
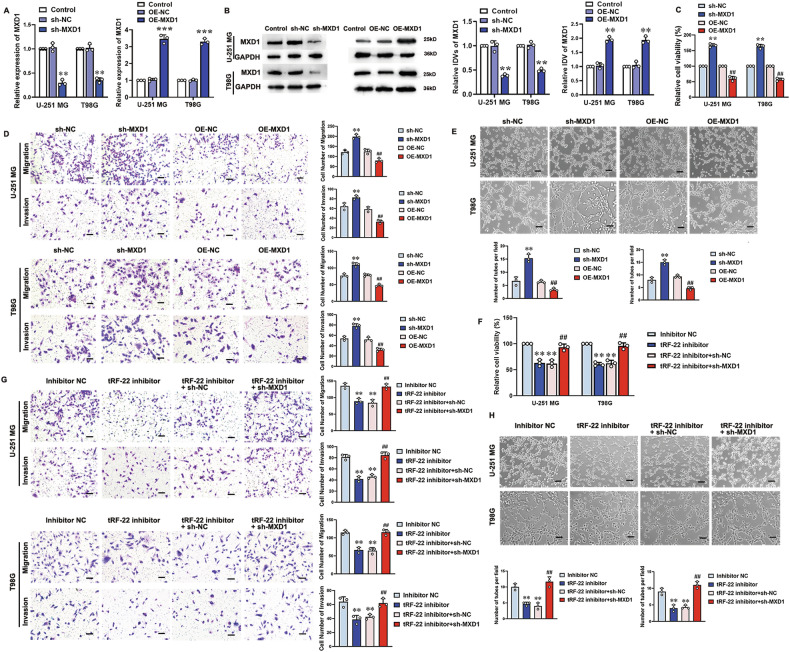
tRF-22 regulates VM formation in GBM by negatively regulating the expression of MXD1. Changes in MXD1 expression were verified by RT-qPCR (**A**) and western blot (**B**), respectively, after silencing or overexpressing MXD1 in U-251 MG and T98G cells. Values are presented as mean ± SD (*n* = 3), **P < 0.01, ***P < 0.001  compared to the NC group. **C** Cell proliferation was assessed using the CCK-8 assay after 48 h of culture (*n* = 5). **D** Cell migration and invasion abilities were evaluated using the Transwell assay after 48 h of culture (*n* = 3). **E** Tube formation ability of cells was assessed using the tube formation assay (*n* = 3). Values are presented as mean ± SD, **P < 0.01 compared to the sh-NC group; ^##^*P* < 0.01 compared to the OE-NC group. Scale bar = 50 μm. **F** After 48 h of transfection with tRF-22 inhibitor in MXD1 stable knockdown U-251 MG and T98G cells, cell proliferation was assessed using the CCK-8 assay (*n* = 5). **G** Cell migration and invasion abilities were evaluated using the Transwell assay (*n* = 3). Scale bar = 50 μm. **H** Tube formation ability of cells was assessed using the tube formation assay (*n* = 3). Values are presented as mean ± SD, **P < 0.01 compared to the inhibitor NC group; ^##^*P* < 0.01 compared to the tRF-22 inhibitor + sh-NC group. Scale bar = 50 μm.

To explore the role of MXD1 in glioma cell function, MXD1 expression was knocked down or overexpressed in U-251 MG and T98G cells, confirmed by RT-qPCR and Western blot (Fig. 5A, B). Cell proliferation, migration, invasion, and tube formation assays showed that MXD1 knockdown significantly increased cell viability (Fig. 5C), migration, invasion (Fig. 5D), and tube formation (Fig. 5E) compared to controls. Conversely, MXD1 overexpression significantly reduced these cell functions (Fig. 5C–E).

To determine whether tRF-22 influences VM formation in glioma cells by negatively regulating MXD1, tRF-22 inhibitor was transfected into U-251 MG and T98G cells with stable MXD1 knockdown. MXD1 knockdown significantly reversed the reductions in cell viability (Fig. 5F), migration, invasion (Fig. 5G), and tube formation (Fig. 5H) induced by tRF-22 inhibitor transfection. These findings suggest that MXD1 downregulation promotes VM formation in glioma cells and that tRF-22 modulates VM formation by negatively regulating MXD1.

### The transcription factor MXD1 binds to the HIF1A promoter region, inhibiting HIF1A transcription

**Fig. 6:**
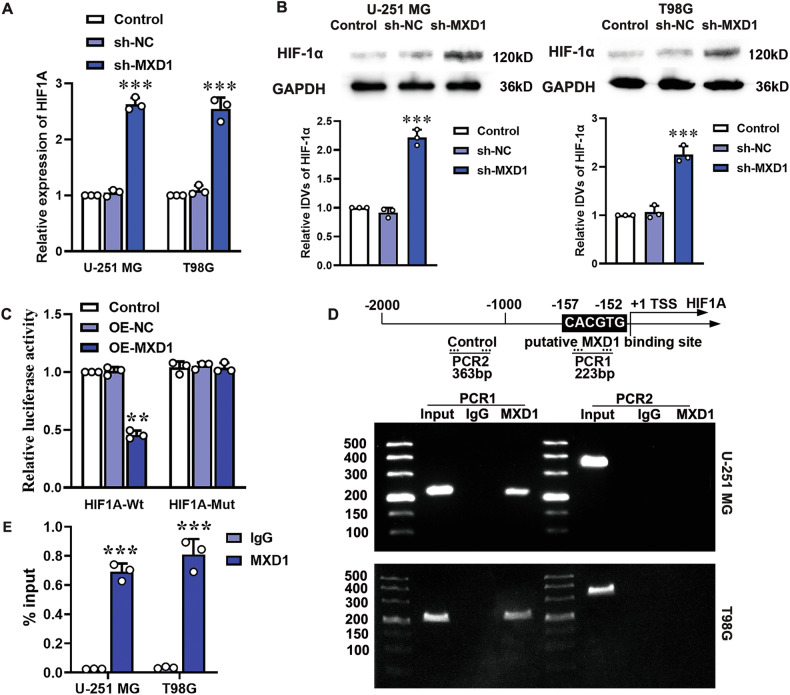
Transcription factor MXD1 binds to the promoter region of the HIF1A gene, inhibiting HIF1A transcription. Changes in HIF-1α expression were detected by RT-qPCR (**A**) and western blot (**B**), respectively, after silencing MXD1 expression in U-251 MG and T98G cells. Values are presented as mean ± SD (*n* = 3), ***P < 0.001 compared to the sh-NC group. **C** The luciferase activity was analyzed using a dual-luciferase reporter system in 293T cells transfected with MXD1 overexpression vector and recombinant luciferase reporter vectors containing either the wild-type or MXD1 binding site mutant promoter region of the HIF1A gene. Data are presented as mean ± SD (*n* = 5), **P < 0.01 compared to the OE-NC group. ChIP (**D**) and ChIP-qPCR (**E**) assay was used to verify the binding site of MXD1 in the promoter region of the HIF1A gene. PCR1 represents the binding site of MXD1 in the promoter region of the HIF1A gene, while PCR2 represents the negative control group without an MXD1 binding site. Data are presented as mean ± SD (*n* = 3), ***P < 0.001 compared to the IgG group.

The effect of MXD1 knockdown on HIF-1α expression was assessed by RT-qPCR and western blot. Compared to the sh-NC group, the MXD1 knockdown group exhibited significantly elevated HIF1A mRNA and protein levels (Fig. 6A, B). Dual-luciferase reporter assays confirmed reduced luciferase activity in the OE-MXD1 and HIF1A 3’UTR-Wt co-transfection group, with no change in the OE-MXD1 and HIF1A 3’UTR-Mut co-transfection group (Fig. 6C). ChIP assays demonstrated that MXD1 binds to the predicted site in the HIF1A promoter region but not to the negative control region (Fig. 6D). ChIP-qPCR assays showed MXD1 was significantly enriched at the HIF1A promoter region (Fig. 6E). These findings suggest MXD1 negatively regulates HIF1A expression by binding to its promoter.

### Independent or combined regulation of TRMT10A and tRF-22 expression effectively inhibits VM formation and tumor growth in nude mice xenografts

**Fig. 7:**
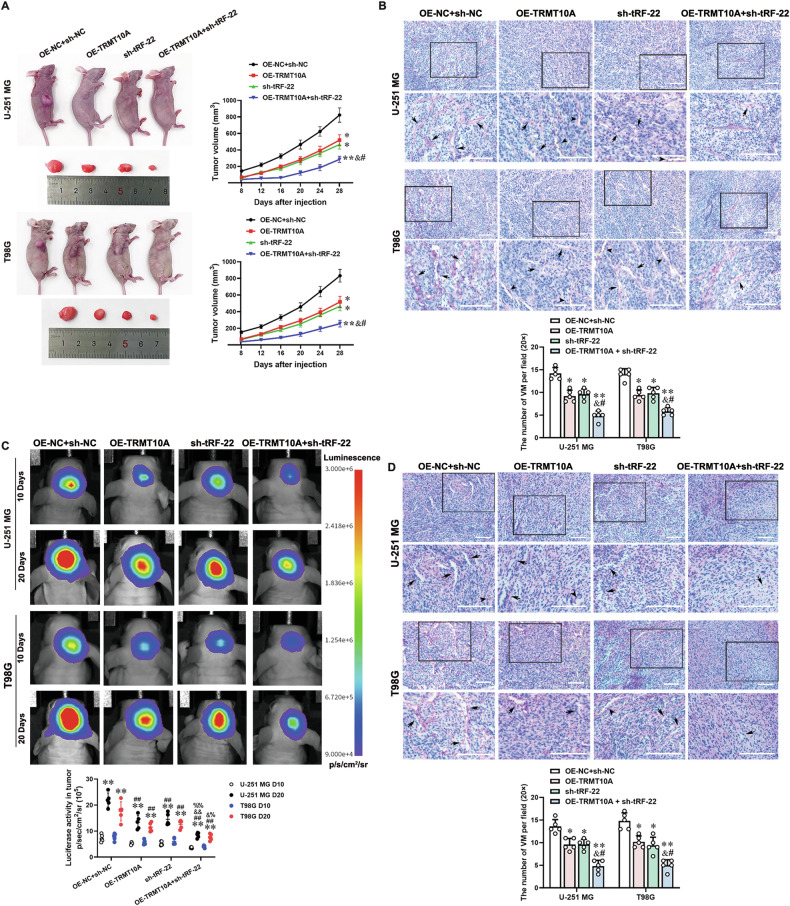
Independent or combined regulation of TRMT10A and tRF-22 expression effectively inhibits VM formation and tumor growth in subcutaneous and orthotopic xenografts in nude mice. **A** Subcutaneous tumor formation and representative tumor samples from each group of nude mice. Data are presented as mean ± SD (*n* = 5). *P < 0.05, **P < 0.01 compared to the OE-NC + sh-NC group; ^#^*P* < 0.05 compared to the OE-TRMT10A group; ^&^*P* < 0.05 compared to the sh-tRF-22 group. CD31/PAS staining was used to detect VM formation in gliomas derived from subcutaneous (**B**) and orthotopic (**D**) xenografts in nude mice. Data are presented as mean ± SD (*n* = 5). *P < 0.05, **P < 0.01 compared to the OE-NC + sh-NC group; ^#^*P* < 0.05 compared to the OE-TRMT10A group; ^&^*P* < 0.05 compared to the sh-tRF-22 group. Scale bar = 100 μm. Black arrows indicate VM, and black arrowheads indicate blood vessels. Enlarged images within black boxes are shown below the respective panels. **C** In vivo bioluminescent imaging of Luc expression of representative tumor‐bearing mice from all groups on days 10 and 20 post‐cell implantation, respectively. Data are presented as mean ± SD (*n* = 5). **P < 0.01 compared to the D10 in each group; ^##^*P* < 0.01 compared to D20 in each OE-NC + sh-NC group; ^&^*P* < 0.05, ^&&^*P* < 0.01 compared to D20 in each sh-tRF-22 group; ^%^*P* < 0.05, ^%%^*P* < 0.01 compared to D20 in each OE-TRMT10A group.

**Fig. 8:**
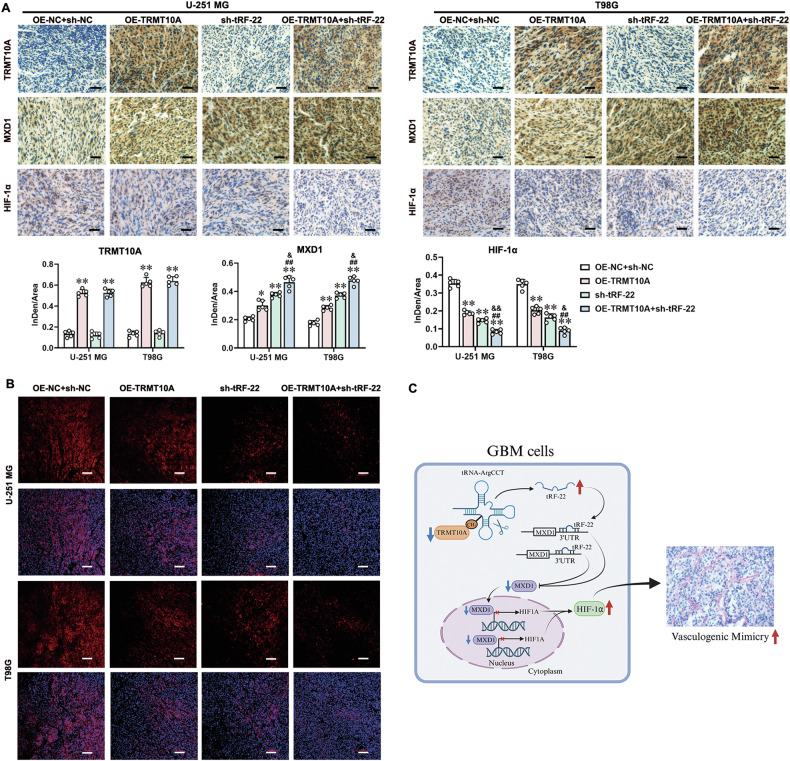
Independent or combined regulation of TRMT10A and tRF-22 expression effectively inhibits VM formation and tumor growth in orthotopic xenografts in nude mice. Immunohistochemical staining for TRMT10A, MXD1, and HIF-1α (**A**), as well as FISH staining for tRF-22 (**B)**, in glioma samples. Data are presented as mean ± SD (*n* = 5). *P < 0.05, **P < 0.01 compared to the OE-NC + sh-NC group; ^##^*P* < 0.01 compared to the OE-TRMT10A group; ^&^*P* < 0.05, ^&&^*P* < 0.01 compared to the sh-tRF-22 group. Scale bar for IHC staining = 200 μm, Scale bar for FISH staining = 100 μm. **C** Schematic diagram of the TRMT10A/tRF-22/MXD1 pathway regulating VM formation in GBM.

The subcutaneous and orthotopic glioma xenograft models in nude mice were used to elucidate the inhibitory effects of the TRMT10A/tRF-22/MXD1 pathway on glioma. Tumor volumes were significantly reduced in the OE-TRMT10A group, the sh-tRF-22 group, and the OE-TRMT10A + sh-tRF-22 group compared to the OE-NC + sh-NC group. Notably, the OE-TRMT10A + sh-tRF-22 group exhibited the smallest tumor volumes (Fig. 7A, C). CD31/PAS double staining showed that VM channels were significantly decreased in the OE-TRMT10A group, the sh-tRF-22 group, and the OE-TRMT10A + sh-tRF-22 group, with the OE-TRMT10A + sh-tRF-22 group having the fewest channels (Fig. 7B, D). IHC and FISH staining of glioma tissue revealed that the expression levels of TRMT10A were significantly elevated in the OE-TRMT10A and OE-TRMT10A + sh-tRF-22 groups compared to the OE-NC + sh-NC group. The expression levels of MXD1 were elevated in the OE-TRMT10A and sh-tRF-22 groups, with the highest level observed in the OE-TRMT10A + sh-tRF-22 group. In contrast, the expression levels of HIF-1α and tRF-22 were significantly reduced in the OE-TRMT10A, sh-tRF-22 and OE-TRMT10A + sh-tRF-22 groups compared to the OE-NC + sh-NC group, with the lowest levels observed in the OE-TRMT10A + sh-tRF-22 group (Fig. 8A, B). These findings suggest that combining TRMT10A overexpression with tRF-22 inhibition significantly reduces the number of VM channels and inhibits tumor growth compared to single treatments.

In summary, both in vitro and in vivo studies confirm that low TRMT10A expression in GBM promotes VM formation by reducing m^1^G9 modification of tRNA-ArgCCT, leading to upregulated tRF-22 expression. tRF-22 binds to and negatively regulates the expression of the transcription factor MXD1, decreasing MXD1’s inhibition of HIF1A transcription, thus promoting VM formation in GBM (Fig. 8C).

## Discussion

Understanding VM formation mechanisms and identifying targeted therapies are crucial for improving GBM outcomes. The aberrant expressions of non-coding RNAs in various tumor tissues are closely associated with VM formation [30–32]. With the advancement of sequencing technologies, increasing evidence indicates that tRNAs, tRFs, and tiRNAs are abnormally expressed in tumor tissues and cells, contributing to cancer development. A key feature of tRNAs is the presence of extensive post-transcriptional modifications. Abnormalities in the expression or function of tRNA-modifying enzymes can affect tRNA structure and stability, leading to various diseases, including cancer [33]. Methylation is one of the most common post-transcriptional modifications of tRNAs, occurring at almost all nitrogen-containing positions of the bases. At position 9, tRNAs can undergo either m^1^G or m^1^A modifications, both of which disrupt G-C or A-U base pairing, altering tRNA structure. The tRNA m^1^G methyltransferase TRMT10A specifically modifies G9; loss of the m^1^G9 modification reduces tRNA stability and increases its cleavage [7, 8]. In this study, TRMT10A expression is downregulated in glioma tissues and cells, correlating with poor prognosis in patients. Downregulation of TRMT10A expression significantly promotes VM formation in vitro, while upregulation of TRMT10A has the opposite effect, suggesting that abnormal TRMT10A expression is associated with VM formation in gliomas. When TRMT10A expression is downregulated, tRF-22 expression is significantly upregulated. Further overexpression of wild-type TRMT10A, but not the catalytically inactive mutant, can downregulate tRF-22. tRF-22 is derived from tRNA-ArgCCT, and its m^1^G9 modification decreases when TRMT10A expression is downregulated. Consistent with our findings, tRNA methyltransferase TRM6/61 is significantly upregulated in GBM, stabilizing tRNA-iMet through m^1^A58 modification and regulating protein translation, thereby promoting GBM development [34, 35].

Research indicates that tRNAs with methylation modifications consistently contain a short variable loop composed of 4-5 nucleotides, while longer variable loops may interfere with substrate binding. The variable loop of tRNA-ArgCCT is composed of 5 nucleotides (positions 44-48), making it more susceptible to methylation [36]. When the G9 position of tRNA-ArgCCT is mutated, downregulation of TRMT10A expression does not alter the level of tRF-22, suggesting that in glioma cells, downregulated TRMT10A increases tRF-22 expression by reducing the m^1^G9 modification of tRNA-ArgCCT, thereby decreasing tRNA stability. Similar to our findings, TRMT10A deficiency in pancreatic β-cells induces tRNA-Gln fragmentation and leads to the accumulation of 5’-tRNA fragments [8]. The m^5^C modifications at specific sites of tRNAs, catalyzed by NSUN2, play a critical role in protecting tRNAs from stress-induced cleavage by angiogenin (ANG), a ribonuclease; however, tRNAs with low levels of modification have increased affinity for ANG, promoting the accumulation of tRFs [37]. Currently, little is known about the generation of tRFs. Existing studies confirm that the production of tRFs involves specific ribonucleases such as ANG and Dicer and is regulated by various stress conditions and tRNA modification states [38]. In this study, the ribonucleases responsible for tRNA-ArgCCT fragmentation remain to be further explored.

tRFs are abnormally expressed in various cancers and can exert either oncogenic or tumor-suppressive regulatory functions. In glioma tissues, tRFs are predominantly i-tRFs in low-grade gliomas and 5’-tRFs in GBM [39]. In this study, tRF-22, classified as an i-tRF, is generated by enzymatic cleavage of the middle region of mature tRNA, spanning multiple contiguous regions of the tRNA. Studies have found that i-tRF-GlyGCC is upregulated in ovarian and colorectal cancers, serving as a biomarker for cancer screening and prognosis [40, 41]. The primary targets of tRFs are mRNAs, followed by various ncRNAs such as lincRNAs and miRNAs, which together account for approximately one-third of their targets. For mRNAs, tRFs mainly target the coding sequence (CDS) and 3’UTR, followed by intronic regions and 5’UTR [42]. Alternatively, tRFs can bind to complementary RNAs to form duplexes, which are recognized and cleaved through classical miRNA mechanisms [43]. For instance, in glioma cells, tRF-19-R118 LOJX targets and negatively regulates the 3’UTR of S100 A11, affecting glioma cell proliferation, migration, and VM formation [39]. Research confirms that the binding motifs or interaction sites of tRFs with RNAs are not restricted by the conventional 2–8 seed region binding, with approximately two-thirds of the motifs located near the 5’ end of the tRF, some extending to the 3’ end, and very few located in the central region [42]. In this study, tRF-22 was predicted to potentially bind to the 3’UTR of MXD1, with the binding motif located near the 5’ end of tRF-22 and extending to the 3’ end, consistent with the findings mentioned above.

Hypoxia is closely associated with VM formation in various tumors. Hypoxia or HIF-1α can promote VM formation in gliomas, lung adenocarcinoma, colorectal cancer, and other cancers [25, 44, 45]. HIF-1α also directly regulates the expression of several VM-related molecules, such as VEGF, Twist, LOX, and MMP2 [46]. The MYC oncogene exerts its transcriptional regulatory functions by forming a heterodimer with MAX; its overexpression promotes the SUMOylation of pVHL, inhibits its ubiquitination, stabilizes HIF-1α, and enhances HIF-1α accumulation [47]. In various tumors, MXD1 functions as a tumor suppressor gene, antagonizing MYC’s transcriptional activity by competing for MAX [22–24]. The MYC/MAX/MAD network is a key signaling pathway implicated in tumor proliferation, cell adhesion, and angiogenesis [48, 49]. In this study, to explore the target genes regulated by tRF-22, we performed RNA-seq analysis to identify differentially expressed genes in U-251 MG cells following the upregulation of tRF-22 expression. Among the significantly downregulated genes, MXD1 showed the most pronounced decrease. MXD1 overexpression in glioma cells markedly suppressed glioma VM formation. Further investigation revealed MXD1 binding sites in the promoter region of the HIF1A gene, confirming that the transcriptional repressor MXD1 can bind to and suppress HIF-1α expression. Finally, in vivo experiments demonstrated that the simultaneous overexpression of TRMT10A and inhibition of tRF-22 significantly reduced glioma size and VM numbers more effectively than either TRMT10A overexpression or tRF-22 inhibition alone.

In conclusion, this study demonstrated that the expression of TRMT10A is downregulated in glioma cells, which reduces m^1^G9 modification of tRNA-ArgCCT, decreases tRNA stability, and upregulates tRF-22 expression. tRF-22 negatively regulates MXD1 expression by binding to the 3’UTR of MXD1 mRNA, thereby diminishing MXD1’s transcriptional repression of the HIF1A gene and promoting glioma VM formation. This study revealed that the TRMT10A/tRF-22/MXD1 pathway plays a critical role in regulating VM formation in GBM, providing new therapeutic targets and strategies for GBM treatment.

### Additional materials and methods

Additional methods are described in Supplementary Methods. Sequences of primers, sh-RNA, tRF-22 mimics, and inhibitors are reported in Supplementary Tables S1–S4.

## Supplementary information

Supplementary Tables

Supplementary Methods

Original western blot figures

## Data Availability

The datasets generated and/or analyzed during the current study are available from the corresponding author on reasonable request.
