# Supplementary material for: TRMT10A regulates tRNA-ArgCCT m1G9 modification to generate tRNA-derived fragments influencing vasculogenic mimicry formation in glioblastoma
Source: Cell Death Dis. 2025 Mar 26;16(1):209. doi: 10.1038/s41419-025-07548-6 (PMC11947273; doi:10.1038/s41419-025-07548-6)
Supplement: Supplementary file 1 — Supplementary Tables [file 41419_2025_7548_MOESM1_ESM.docx]

Supplemental Table 1 shRNA sequences

| Name | Target sequence |
| --- | --- |
| sh-TRMT10A  sh-MXD1  sh-tRF-22  sh-NC | CAGAGCACUAUAGUGAACUCAUAAA  GCACCAGCAUCAAGAGAAUAA  UCCUAAGCCAGGGAUUGUGGG  UUCUCCGAACGUGUCACG |

Supplemental Table 2 tRF-22 mimic and inhibitor sequences

| Name | Sequence (5’ to 3’) |
| --- | --- |
| tRF-22 mimic  tRF-22 mimic NC  tRF-22 inhibitor  tRF-22 inhibitor NC | UCCUAAGCCAGGGAUUGUGGGU  UUCUCCGAACGUGUCACGUTT  ACCCACAAUCCCUGGCUUAGGA  ACGUGACACGUUCGGAGAATT |

Supplemental Table 3 Primers used for qRT-PCR

| Gene Name | Primers (5’ to 3’) |
| --- | --- |
| TRMT10A  MXD1  GAPDH  U6  tRF-22  HIF1A  tRNA-ArgCCT  Adaptor | Forward 5’-CGATGTTACGCAGAAAACCGACG-3’  Reverse 5’-CCTTCCAGTTGACCCATCCTTTG-3’  Forward 5’-ACCTGAAGAGGCAGCTGGAGAA-3’  Reverse 5’-AGATAGTCCGTGCTCTCCACGT-3’  Forward 5’-GGACCTGACCTGCCGTCTAG-3’  Reverse 5’-TAGCCCAGGATGCCCTTGAG-3’  Forward 5’-GCTTCGGCACATATACTAAAAT-3’  Reverse 5’-CGCTTCACGAATTTGCGTGTCAT-3’  Forward 5’-TCCTAAGCCAGGGATTGTGGGT-3’  Reverse 5’-AACGCTTCACGAATTTGCGT-3’  Forward 5’-TATGAGCCAGAAGAACTTTTAGGC-3’  Reverse 5’-CACCTCTTTTGGCAAGCATCCTG-3’  Forward 5’-TAAGCCAGGGATTGTGGGTTC-3’  Reverse 5’-AGGCCARTGCCTTATCCATTAGG-3’  5’phos-TCGTAGGGTCCGAGGTATTCACGAT/rGrGU/-3’ |

Supplemental Table 4 Antibodies used for western blot, dot blot, ChIP, IF and IHC

| Protein | Application | Antibody | Origin | Dilution | Observed  Molecular weight |
| --- | --- | --- | --- | --- | --- |
| TRMT10A | WB | 17294-1-AP, Proteintech | Rabbit | WB: 1:1000 | 40kDa |
| TRMT10A | IF, IHC | NBP2-55473, Novus | Rabbit | IF: 1:100, IHC: 1:150 |  |
| MXD1 | WB, ChIP | 19547-1-AP, Proteintech | Rabbit | WB: 1:1000, ChIP: 5 μg for 5 μg chromatin | 25kDa |
| MXD1 | IHC | PA5-51609, Invitrogen | Rabbit | IHC: 1:300 |  |
| HIF-1α | WB, IHC | 20960-1-AP, Proteintech | Rabbit | WB: 1:1000, IHC: 1:150 | 120kDa |
| GAPDH | WB | 60004-1-ig, Proteintech | Rabbit | WB: 1:100000 | 36kDa |
| anti-m^1^G | Dot Blot | EPR19833-150, Abcam | Rabbit | Dot: 1:1000 |  |

Supplemental Table 5 Primers used for ChIP-PCR

| Gene name | Binding site | Primer (5’ to 3’) |
| --- | --- | --- |
| HIF1A  HIF1A control | PCR-1  PCR-2 | Forward 5’-GCCGAGGAGAAAGAGAGCAGGAG-3’  Reverse 5’-CGCTCACGTGCTCGTCTGTGTTTA-3’  Forward 5’-ACGACTTGCCAGTAAGATTC-3’  Reverse 5’-TTAGCGGACAGGGTGGTT-3’ |

Supplemental Table 6 Primers used for ChIP-qPCR

| Gene name | Binding site | Primer (5’ to 3’) |
| --- | --- | --- |
| HIF1A  HIF1A control | PCR-1  PCR-2 | Forward 5’-GCCGAGGAGAAAGAGAGCAGGAG-3’  Reverse 5’-CGCTCACGTGCTCGTCTGTGTTTA-3’  Forward 5’-ACGACTTGCCAGTAAGATTC-3’  Reverse 5’-GGGAAATGCCTGAAGAG-3’ |
