# Supplementary material for: TRMT10A regulates tRNA-ArgCCT m1G9 modification to generate tRNA-derived fragments influencing vasculogenic mimicry formation in glioblastoma
Source: Cell Death Dis. 2025 Mar 26;16(1):209. doi: 10.1038/s41419-025-07548-6 (PMC11947273; doi:10.1038/s41419-025-07548-6)
Supplement: Supplementary file 2 — Supplementary Methods [file 41419_2025_7548_MOESM2_ESM.docx]

**SUPPLEMENTARY MATERIALS AND METHODS**

**Patient Tissue Samples**

Glioma tissue specimens were obtained from patients at Shengjing Hospital of China Medical University, diagnosed according to WHO criteria. The study was approved by the Shengjing Hospital Ethics Committee (2020PS104K) and adhered to the Helsinki convention. Informed consent was obtained from all patients. Fresh glioma samples and non-neoplastic brain tissues (from temporal lobectomy for epilepsy) were snap-frozen in liquid nitrogen immediately upon removal. Portions of each sample were formalin-fixed, paraffin-embedded, and stored at room temperature.

**Cell Culture**

Human brain astrocytes (SVG P12) and human glioma cells (T98G) were cultured in MEM medium supplemented with 10% fetal bovine serum (FBS). Human glioma cells (U-87 MG and U-251 MG) were cultured in high-glucose DMEM medium supplemented with 10% FBS. All the cell lines were verified by short tandem repeat (STR) analysis and eliminated mycoplasma contamination. All cells were maintained at 37°C in a humidified incubator with 5% CO_2_.

**Cell Transfection**

The overexpression plasmids for TRMT10A and MXD1 and their corresponding negative control (NC) were constructed by Syngentech (Beijing, China). The knockdown vectors for TRMT10A and MXD1, the scramble control (sh-NC), and the overexpression vector for tRNA-ArgCCT were constructed by GeneChem (Shanghai, China). The tRF-22 mimics, inhibitors and NC were designed and synthesized by GenePharma (Shanghai, China). U-251 MG and T98G cells were transfected using Opti-MEM and Lipofectamine 3000 transfection reagent (Thermo Fisher Scientific, CA, USA) according to the manufacturer’s instructions. The sh-tRF-22 lentivirus, obtained from GeneChem (Shanghai, China), was used to infect the cells in the presence of polybrene. To obtain the stable cell lines, puromycin (Solarbio, China) was added to the cells 48 h post-transfection or infection. All the sequences are shown in Supplementary Table 1 and 2.

**Quantitative Real-time PCR (qRT-PCR)**

Total RNA was extracted from cells using Trizol reagent (Life Technologies, CA, USA) according to the manufacturer’s instructions. qRT-PCR was performed using the PrimeScript™ II 1st Strand cDNA Synthesis Kit and TB Green® Premix Ex Taq™ II (Tli RNaseH Plus, Takara, Japan). The Ct values were determined, and the relative expression levels of the target genes were calculated using the 2^-ΔΔ^Ct method with GAPDH as the internal reference. The tRNA-ArgCCT was detected as previously described[29]. Briefly, the extracted RNA was deacylated using 20 mmol/L Tris-HCl (pH 9.0) at 37°C for 40 minutes, followed by RNA purification. The RNA was then ligated with 20 pmol/L U-adaptor using 0.1 μL T4 RNA ligase and 2 μL ligase buffer in a 20 μL ligation reaction. After ligation, the resultant RNA was incubated with 1 μL tRNA-ArgCCT reverse primers (10 pmol) at 65°C for 5 minutes, followed by reverse transcription. The resultant cDNA was subjected to qPCR. Small RNA was extracted using a miRNA extraction kit (Vazyme, Nanjing, China), deacylated with the rtStar™ tRF&tiRNA pretreatment kit (Aksomics, Shanghai, China), and reverse transcribed using a miRNA polyA tailing kit (Sangon, Shanghai, China). The primers used above are listed in Supplementary Table 3.

**Western Blot**

Cells were lysed with RIPA buffer (Epizyme, China) supplemented with protease inhibitors (Beyotime, China). After sonication, the lysates were centrifuged at 17000 g for 40 minutes at 4°C. The protein concentration was determined using a BCA Protein Assay Kit (Beyotime, China). Equal amounts of protein were subjected to SDS-PAGE electrophoresis. After electrophoresis, the proteins were transferred to a PVDF membrane, blocked with 5% non-fat milk in Tris-buffered saline/Tween 20 (TBST) at room temperature for 2 h. The PVDF membrane was then incubated with the primary antibodies overnight at 4°C. The primary antibodies used in this study are listed in Supplementary Table 4. After incubation with the respective secondary antibodies for 2 h at room temperature, the membrane was washed with TBST and then detected using an enhanced chemiluminescence detection kit (Beyotime, China). The bands were visualized using the automatic chemiluminescence image analysis system (Tanon, China). The results were quantified using Image J software, and the integrated density values (IDV) of the target protein were normalized to GAPDH expression.

**Chromatin Immunoprecipitation (ChIP) Assay**

ChIP analysis was performed using a Chromatin Immunoprecipitation Assay Kit (Cell Signaling Technology, MA, USA). Cells were fixed with formaldehyde and then chromatin was fully digested with nucleases. The cell lysates were sonicated, and DNA purified. A portion of the sonicated lysate served as Input positive control, while the rest was divided and immunoprecipitated with anti-MXD1 antibody or IgG antibody overnight at 4°C. Protein-DNA complexes were then incubated with Protein A/G beads at 4°C for 2 h, followed by multiple washes and DNA elution. cDNA was obtained according to the instructions of the PrimeScript™ II 1st Strand cDNA Synthesis Kit and subjected to reverse transcription PCR (RT-PCR) using PrimeSTAR® Max DNA Polymerase. The resulting samples were electrophoresed on a 3% agarose gel at 100 V and 40 mA for 45 minutes, and images were captured using a gel imaging system. The ChIP-qPCR amplification was performed following the qPCR protocol as described above. The antibody and primer sequences used are shown in Supplementary Table 4-6.

**Nuclear and Cytoplasmic RNA Extraction**

According to the instructions provided with the nuclear-cytoplasmic separation kit (Norgen Biotek, Canada), Buffer J, Buffer SK, and Wash Solution A were prepared. Briefly, Buffer J was added to the cells and centrifuged. The supernatant, containing the cytoplasmic RNA, was transferred to a new RNase-free centrifuge tube, while the pellet contained the nuclear RNA. To the cytoplasmic or nuclear RNA tube, Buffer SK and 100% ethanol were added to centrifuge. After washing, Buffer E was added to elute the nuclear and cytoplasmic RNA.

**Dual-Luciferase Reporter Assay**

The MXD1 3’UTR wild-type and tRF-22 binding site mutant sequences were inserted into the pGL3-Basic-Luciferase reporter vector to construct the MXD1 3’UTR wild-type (MXD1 3’UTR-Wt) and mutant (MXD1 3’UTR-Mut) luciferase expression vectors. HEK293T cells were co-transfected with the recombinant luciferase expression vectors and tRF-22 mimic using Lipofectamine 3000. Similarly, the HIF1A promoter region wild-type sequence and the MXD1 binding site mutant sequence were inserted into the pGL3-Basic-Luciferase reporter vector to construct the HIF1A wild-type (HIF1A-Wt) and mutant (HIF1A-Mut) luciferase expression vectors. HEK293T cells were co-transfected with the recombinant luciferase expression vectors and MXD1 overexpression vector using Lipofectamine 3000. After 48 h, the Dual-Luciferase Reporter Assay System (Promega, WI, USA) was used to measure the activities of firefly luciferase and Renilla luciferase. The relative luciferase activity was normalized by Renilla luciferase activity.

**RNA Pull-Down Assay**

For specific isolation of tRNA-ArgCCT, 50 μL of magnetic beads (Dynabeads™ MyOne™ Streptavidin C1, Invitrogen, USA) were washed three times with 1× B&W washing buffer, followed by two washes with Solution A and one wash with Solution B, according to the manufacturer's instructions. The magnetic beads were resuspended in 50 μL of 6× NTE solution (20× NTE solution containing 4 M NaCl, 0.4 M Tris-HCl pH 7.5, and 50 mM EDTA) with RNase inhibitor added. Then, 200 pmol of biotin-labeled tRNA-ArgCCT probe was added to the bead suspension, mixed gently, and incubated at room temperature for 30 minutes. After incubation, the beads were washed three times with 1× B&W buffer to remove unbound biotin probes. The beads were then resuspended in 12× NTE solution with RNase inhibitor added, and an equal volume of RNA was added. The bead-RNA mixture was heated at 70°C for 30 minutes, followed by slow cooling to 30°C, and incubated gently for 4 h. After separation of the beads, they were washed three times with 3× NTE, two times with 1× NTE, and once with 0.1× NTE. The RNA was eluted from the beads with 10 μL of RNase-free water at 70°C for 5 minutes. The concentration of tRNA was measured, and the samples were enzymatically digested into nucleosides, followed by dephosphorylation. The pretreated nucleoside solution was deproteinized using a 10,000-Da MWCO spin filter. The nucleoside mixtures were analyzed using an Agilent 6460 QQQ mass spectrometer with an Agilent 1260 HPLC system (Agilent Technologies, USA). LC-MS data of m^1^G were acquired using Agilent Qualitative Analysis software.

**Dot Blot Analysis**

RNA concentration was measured after RNA extraction. RNA samples were diluted in a concentration gradient (800, 400, 200, 100 ng/μL), heated at 95°C for 3 minutes, and then cooled on ice. After mixing, 2 μL of each RNA sample was spotted onto a nylon membrane (0.45 μm, Beyotime, China). After air-drying the membrane, it was UV-crosslinked (254 nm) for 40 minutes. The membrane was washed with TBST and then incubated in blocking buffer (TBST containing 5% non-fat milk) at room temperature for 1 hour. The membrane was then incubated overnight at 4°C with anti-m^1^G antibody. After washing, the membrane was incubated with secondary anti-rabbit antibody at room temperature for 1 hour. The membrane was scanned using the Tanon automatic chemiluminescence image analysis system after ECL reagent treatment. The membrane was then transferred to methylene blue staining solution and incubated at room temperature for 30 minutes, followed by scanning using an image analysis system. The primary antibodies used are listed in Supplementary Table 4.

**RNA Fluorescence In Situ Hybridization (FISH)**

Cells were seeded in glass bottom dish and cultured overnight. Following the instructions of the FISH kit for RNA, cells were treated with 0.1% Buffer A (containing Triton-X-100) at room temperature for 15 minutes, washed and incubated at 37°C for 30 minutes. Subsequently, 2× Buffer C (20× SSC) was added to each dish, and the incubation was continued at 37°C for 30 minutes. Denatured probes were then added to the dishes, which were incubated in the dark at 37°C overnight for hybridization. The following day, the dishes were washed with 0.1% Buffer F (containing Tween 20) and 2× Buffer C. DAPI staining was performed in the dark for 20 minutes, followed by observation under a laser confocal microscope (Nikon AX R, USA).

**Immunofluorescence (IF)**

The cells seed in glass bottom dish were fixed using 4% paraformaldehyde for 30 min, permeabilized with 0.3% Triton X-100 for 5 min, blocked with 5% BSA for 2 h, and incubated with anti-TRMT10A primary antibody at 4℃ overnight. On the next day, the dishes were washed using PBST and the cells were incubated with the secondary antibodies at room temperature in dark for 2 h. The nuclei were stained using DAPI for 5 min, and the dishes were sealed with an anti-fluorescence quencher, observed, and photographed under a laser confocal microscope (Nikon AX R, USA). The primary antibodies used are listed in Supplementary Table 4.

**Cell Migration Assay**

The U-251 MG cells and T98G cells were transfected at concentrations of 1×10^4^ and 8000 cells/mL, respectively, were seeded into the upper chamber of a 24-well Transwell insert. Meanwhile, 500 μL of medium containing 10% FBS was added to the lower chamber. The cells were then incubated at 37°C with 5% CO_2_ for 48 h. After incubation, the Transwell inserts were washed, and fixed with 4% paraformaldehyde for 20 minutes, stained with crystal violet for 5 minutes, and then stained with Giemsa solution overnight. The next day, after washing with PBS, cells were observed under a microscope (Olympus, Tokyo, Japan).

**Cell Invasion Assay**

For the cell invasion assay, Matrigel was diluted 1:8 with serum-free medium at 4°C. Subsequently, 50 μL of the diluted Matrigel was evenly coated on the upper surface of the Transwell insert. The inserts were then incubated at 37°C for 30 minutes to allow Matrigel hydration. The subsequent experimental procedure was the same as the cell migration assay.

**Tube Formation Assay**

After overnight melting at 4°C, 300 μL of Matrigel was added to each well of a pre-cooled 24-well plate. The plate was then incubated at 4°C and 37°C for 30 minutes each. Subsequently, the transfected U-251 MG and T98G cells, at concentrations of 2×10^5^ and 1×10^5^ cells per well, respectively, were seeded onto the Matrigel surface and incubated at 37°C with 5% CO_2_ for 3-4 h. Finally, tube-like structures formed by the cells were observed under a microscope (Olympus, Tokyo, Japan).

**Functional Enrichment Analysis of the Differential Expressed genes regulated by tRF-22 mimic**

The differential expressed genes regulated by tRF-22 mimic were subjected to Gene Ontology (GO) analysis implemented by Metascape (https://metascape.org/) database. Only terms with a *p*-value < 0.05, a minimum count of 3, and an enrichment factor >1.5 were collected and grouped into clusters based on their membership similarities.

**Subcutaneous and** **orthotopic Xenograft Tumor Model in Nude Mice**

Six-week-old BALB/c female nude mice were obtained from Huafukang Company (Beijing, China) and divided into the following four groups: OE-NC + sh-NC, OE-TRMT10A, sh-tRF-22, OE-TRMT10A + sh-tRF-22, with 5 mice in each group. For subcutaneous xenograft tumor model, prior to transplantation, the right axilla of each nude mouse was disinfected, and 6×10^5^ cells were slowly injected subcutaneously into the right axilla. The mice were observed for tumor formation, and 30 days after transplantation, the mice were euthanized to obtain tumor tissues. Tumor size was measured and photographed. Tumor volume was calculated using the following formula: Volume (mm^3^) = (length × width^2^) / 2. For the orthotopic xenograft tumor model, glioma cells stably overexpressing TRMT10A or with reduced expression of tRF-22 were labeled with the Luc reporter gene using a lentiviral vector. 5 × 10^4^ cells in 3 µL of PBS were stereotactically injected into the right striatum of 6-week-old BALB/c nude mice (coordinates relative to the bregma: medial-lateral +2 mm, anterior-posterior +1 mm and dorsal-ventral −3 mm). Tumor growth was monitored using an in vivo imaging system (SkyView, China). A region of interest was defined over the tumors to quantify signal intensities recorded as total photon counts per second per cm^2^ (photons/sec/cm^2^/sr). All nude mouse experiments were conducted strictly in accordance with the approved protocol by the Ethics Committee of China Medical University (KT2022330).

**CD31-PAS and IHC Staining**

Fresh tissue specimens fixed with 4% paraformaldehyde were embedded in paraffin, sectioned into 5 μm thick slices, and subjected to deparaffinization, hydration, and antigen retrieval, followed by staining using an immunohistochemical staining kit (Maxim, China). After the peroxidase and goat serum blocking steps, the sections were incubated with CD31 antibody (Proteintech, 11265-1-AP, 1:3000) overnight at 4°C. The next day, after secondary antibody incubation, the sections were processed for DAB staining and observed under a microscope. Subsequently, the sections were subjected to periodic acid‑schiff staining (PAS) using iodine solution and Schiff reagent (BaSO, China), followed by nuclear staining with hematoxylin. For the IHC staining of TRMT10A, MXD1, and HIF-1α, tumor sections were incubated overnight at 4 °C with primary antibodies. The following day, sections were exposed to biotin-conjugated secondary antibodies at 37 °C for 1 hour, visualized with diaminobenzidine, and counterstained with hematoxylin. After dehydration and clearing, the sections were mounted with neutral resin and air-dried. Finally, tissue sections were observed under a microscope. The primary antibodies used are listed in Supplementary Table 4.
