# Supplementary material for: TRMT10A regulates tRNA-ArgCCT m1G9 modification to generate tRNA-derived fragments influencing vasculogenic mimicry formation in glioblastoma
Source: Cell Death Dis. 2025 Mar 26;16(1):209. doi: 10.1038/s41419-025-07548-6 (PMC11947273; doi:10.1038/s41419-025-07548-6)

Supplementary Fig. 1 Original western blots of Figure 1E.

TRMT10A

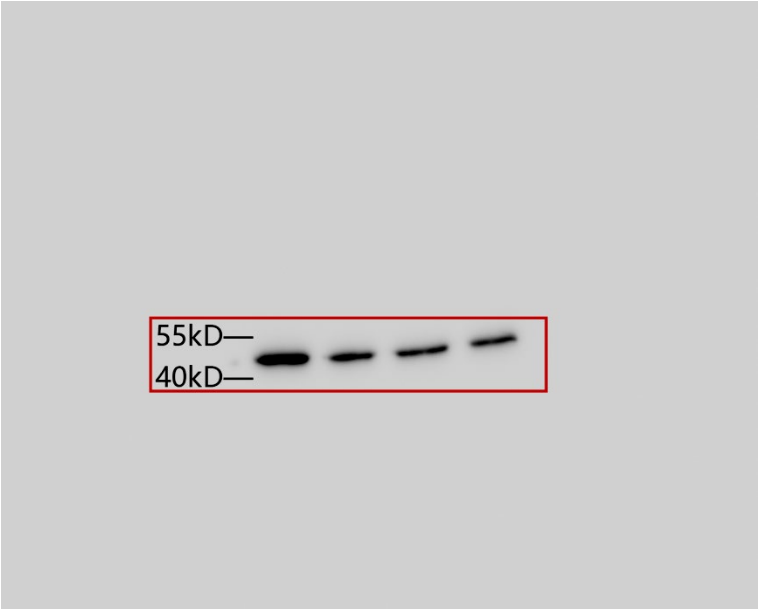

GAPDH

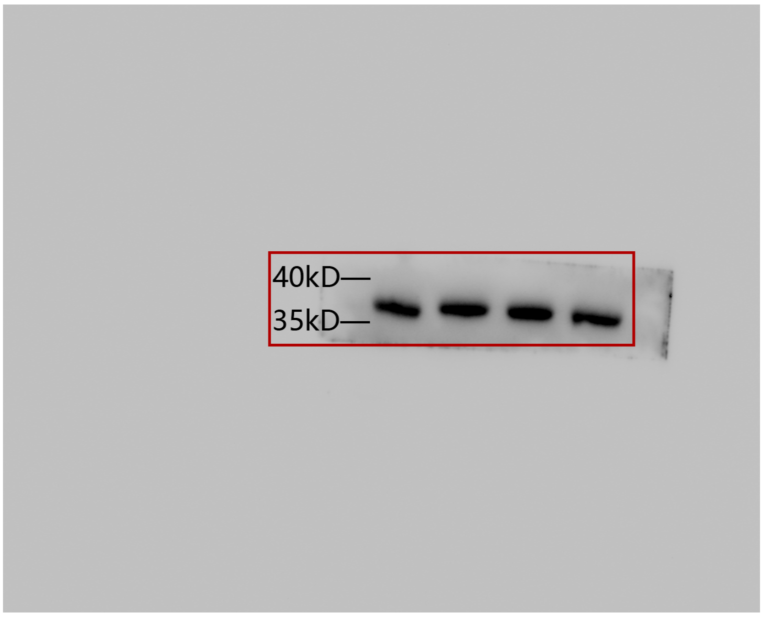

Supplementary Fig. 2 Original western blots of Figure 1G.

U-251 MG **T98G**

TRMT10A

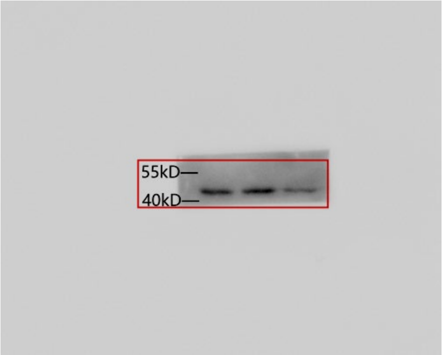

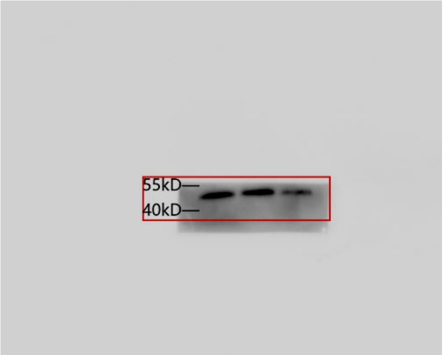

GAPDH

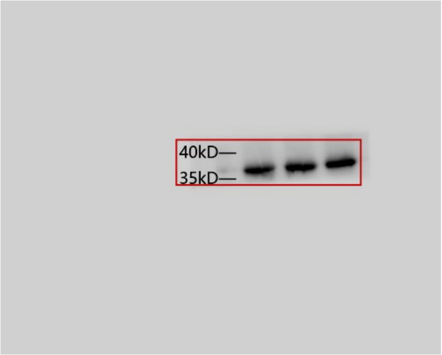

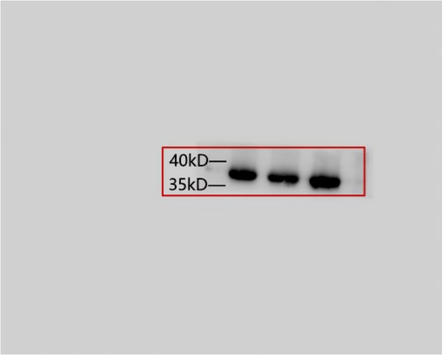

Supplementary Fig. 3 Original western blots of Figure 1M.

U-251 MG | T98G

TRMT10A

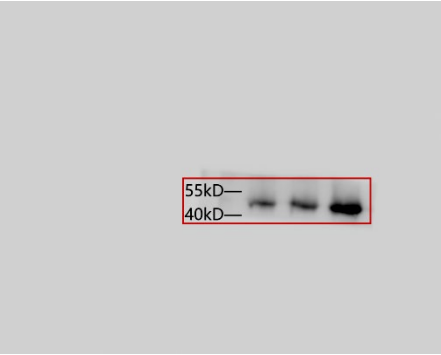

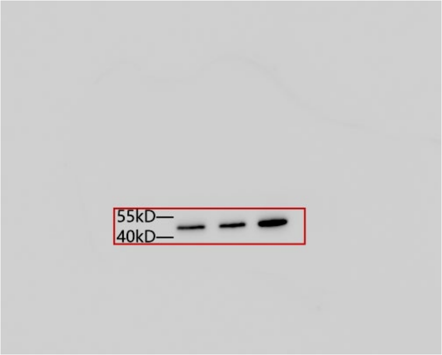

GAPDH

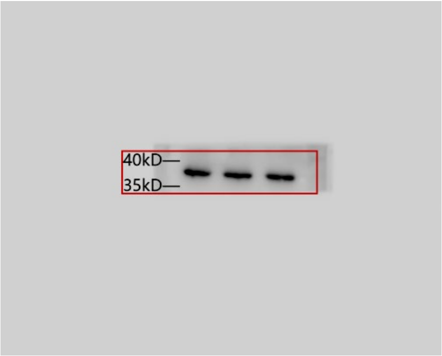

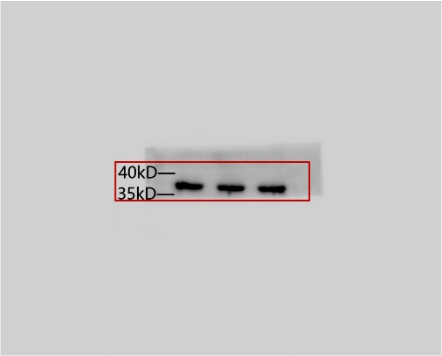

Supplementary Fig. 4 Original western blots of Figure 4G.

GAPDH

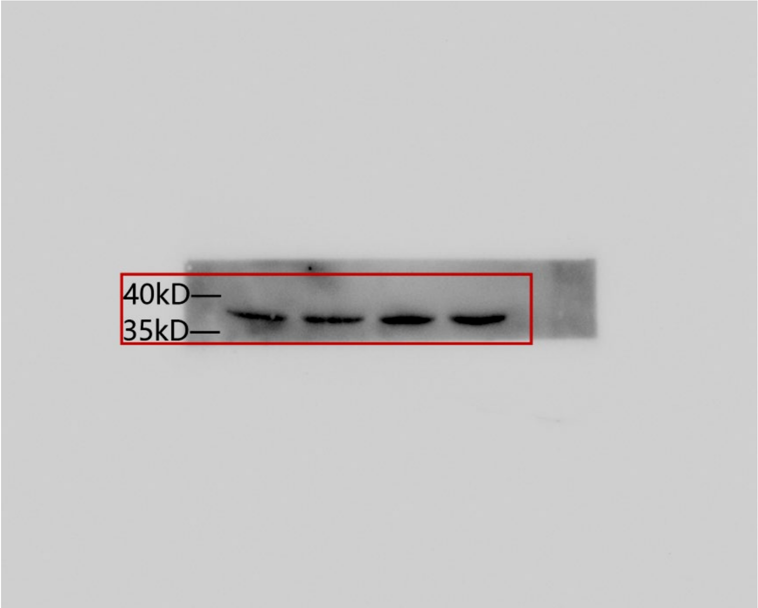

MXD1

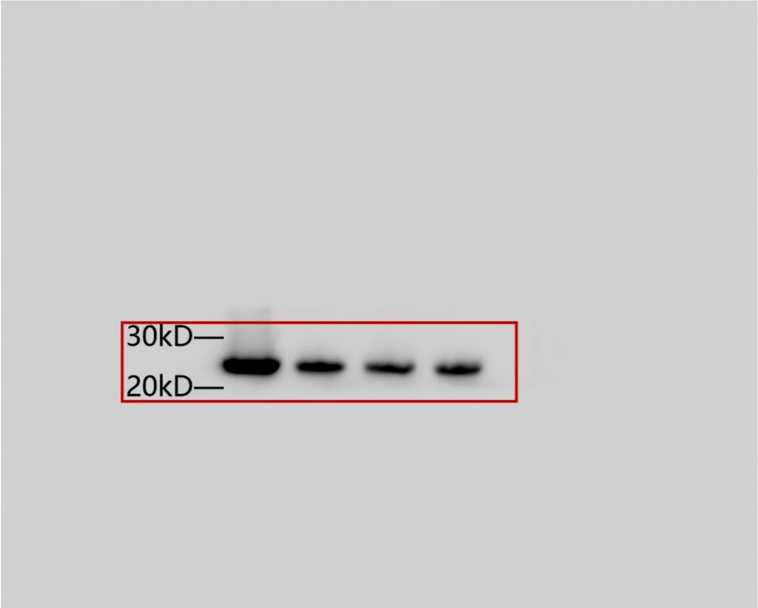

Supplementary Fig. 5 Original western blots of Figure 4I (left panel).

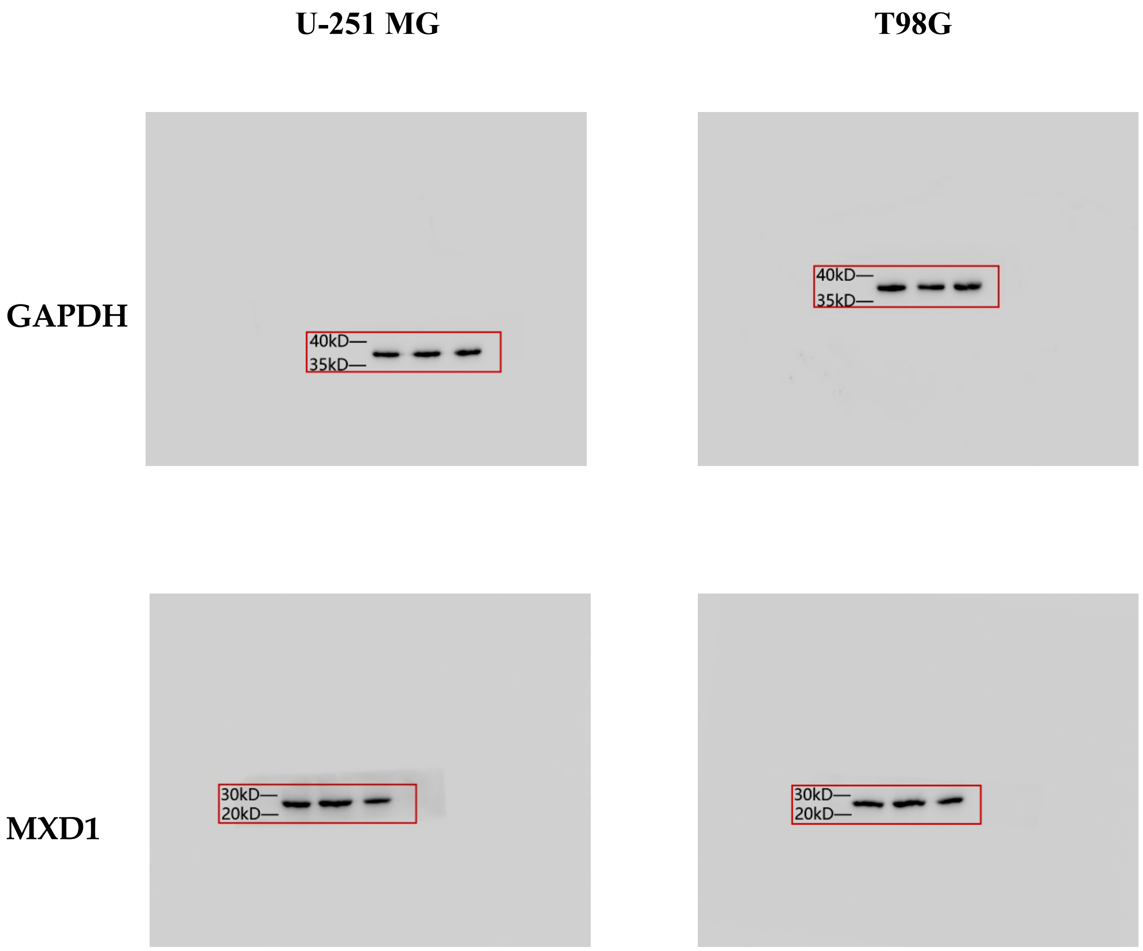

Supplementary Fig. 6 Original western blots of Figure 4I (right panel).

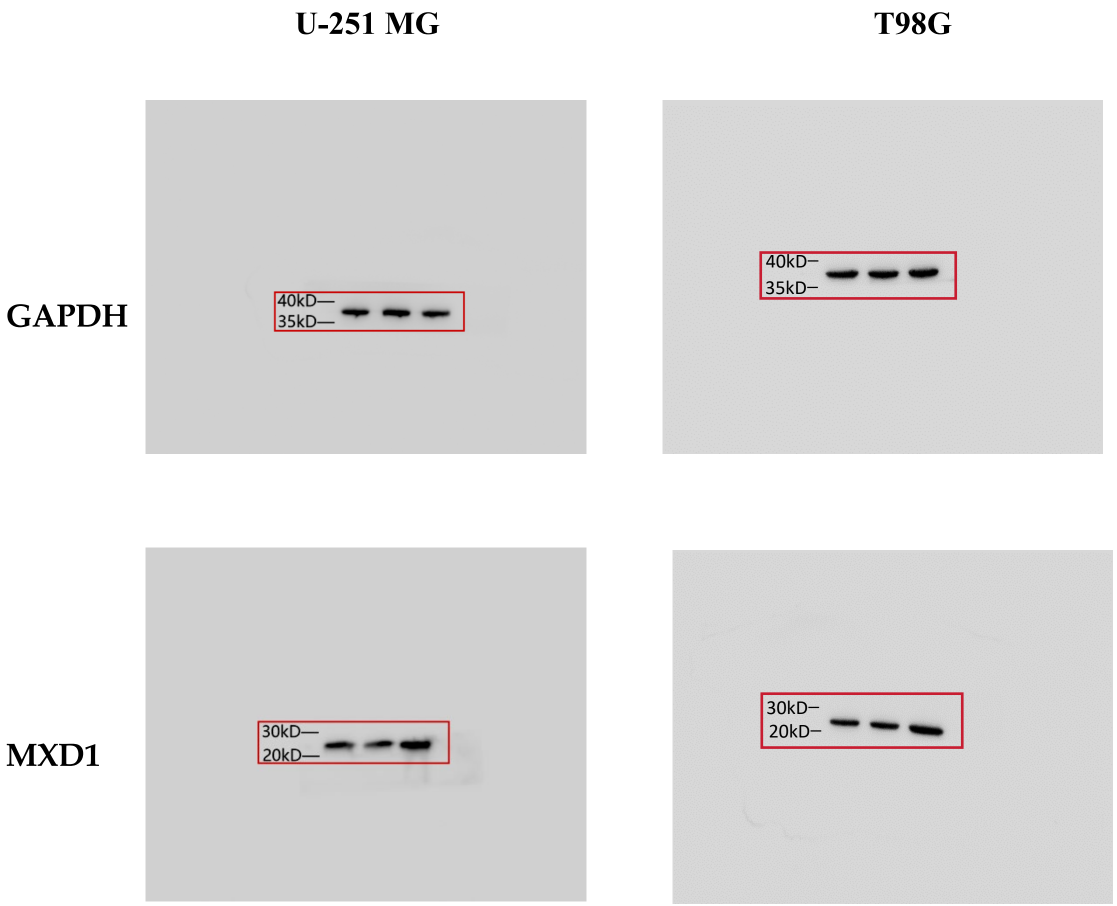

Supplementary Fig. 7 Original western blots of Figure 5B (left panel).

U-251 MG

T98G

GAPDH

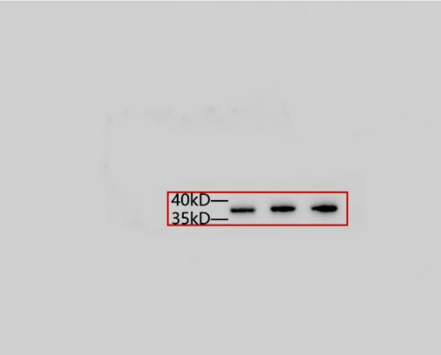

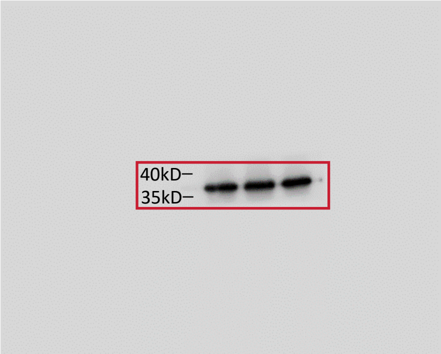

MXD1

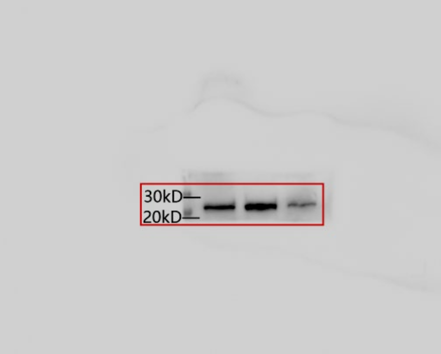

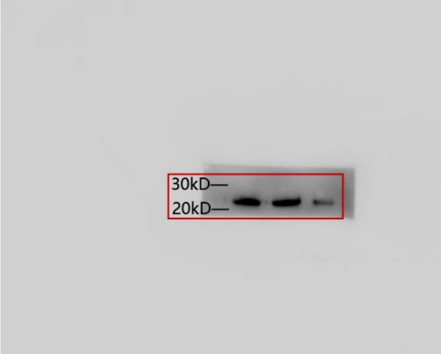

Supplementary Fig. 8 Original western blots of Figure 5B (right panel).

U-251 MG **T98G**

GAPDH

MXD1

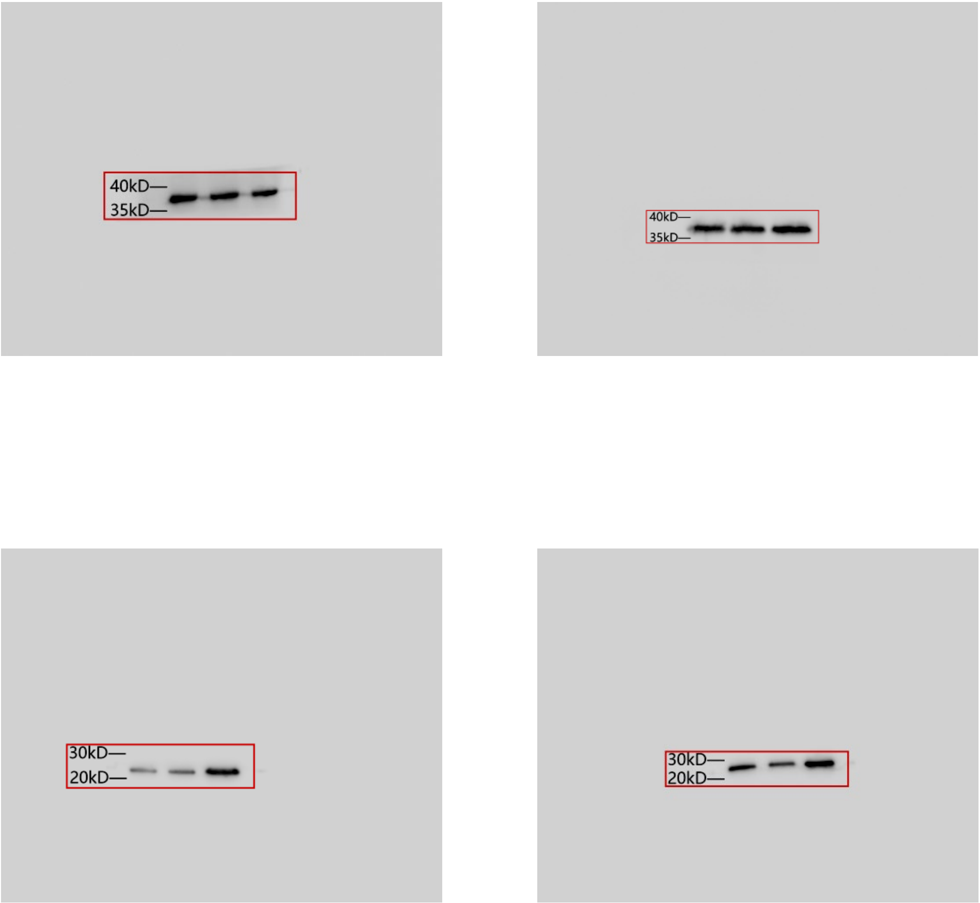

Supplementary Fig. 9 Original western blots of Figure 6B.

U-251 MG　　　　T98G

HIF-1 α

GAPDH

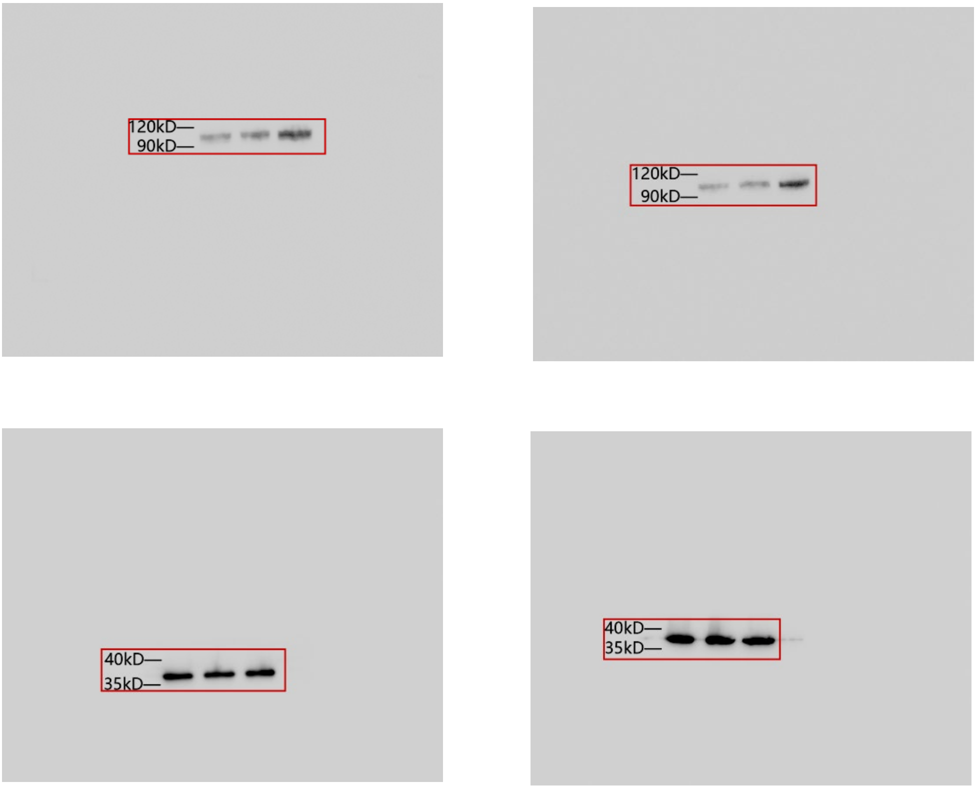

Supplement: Supplementary file 3 — Original western blot figures [file 41419_2025_7548_MOESM3_ESM.pdf]
